# Benazepril affects integrin-linked kinase and smooth muscle α-actin expression in diabetic rat glomerulus and cultured mesangial cells

**DOI:** 10.1186/1471-2369-15-135

**Published:** 2014-08-20

**Authors:** Honglin Niu, Lei Nie, Maodong Liu, Yanqing Chi, Tao Zhang, Ying Li

**Affiliations:** 1 Department of Nephrology, The Third Hospital of Hebei Medical University, Shijiazhuang 050051, China; 2 Key Laboratory of Kidney Diseases of Hebei Province, Shijiazhuang 050071, China; 3 Cardiovascular Molecular Imaging Laboratory, Section of Cardiovascular Medicine, Yale University, New Haven, Connecticut 06511, USA; 4 VA Connecticut Healthcare System, West Haven, Connecticut 06519, USA

**Keywords:** Diabetic nephropathy, Integrin-linked kinase, Smooth muscle α-actin, Benazepril, Glomerular mesangial cells

## Abstract

**Background:**

Diabetic nephropathy (DN) is the leading cause of chronic kidney disease and is associated with excessive cardiovascular morbidity and mortality. The angiotensin converting enzyme inhibitor (ACEI) benazepril has been shown to slow the progression of chronic renal disease and have beneficial effects in patients with a combination of chronic renal disease and cardiovascular disease. Transforming growth factor-β_1_ (TGF-β_1_) plays a central role in the pathogenesis and progression of DN. Integrin-linked kinase (ILK) can modulate TGF-β_1_-induced glomerular mesangial cell (GMC) injury, which is a prominent characteristic of renal pathology in kidney diseases. As an integrin cytoplasmic-binding protein, ILK regulates fibronectin (FN) matrix deposition and the actin cytoskeleton. Smooth muscle α-actin (α-SMA) is involved in progressive renal dysfunction in both human and experimental renal disease.

**Methods:**

To explore the mechanisms of benazepril’s reno-protective effects, we examined the expression of TGF-β_1_, ILK, and α-SMA in GMC exposed to high glucose (HG) and in the kidneys of streptozotocin (STZ)-induced diabetic rats using real-time quantitative RT-PCR and western blot analysis. To elucidate the mechanism(s) of the effect of benazepril on GMC cellular processes, we assessed the effect of benazepril on Angiotensin II (Ang II) signalling pathways using western blot analysis.

**Results:**

The expression of TGF-β_1_, ILK, and α-SMA increased significantly in the diabetic group compared with the control group. Benazepril treatment inhibited the expression of these genes in DN but failed to rescue the same levels in the control group. Similar results were found in GMC treated with HG or benazepril. Ang II increased ERK and Akt phosphorylation in the HG group, and benazepril could not completely block these responses, suggesting that other molecules might be involved in the progression of DN. Our findings suggest that benazepril decreases ILK and α-SMA expression, at least in part, by affecting the interactions between Ang II and TGF-β_1_.

**Conclusions:**

The findings described here support the hypothesis that the HG milieu of diabetes increases TGF-β_1_ secretion, which increases the synthesis of ILK and α-SMA that are involved in the progression of DN. This might be an important mechanism of the benazepril renal-protective function in the pathogenesis of DN.

## Background

Diabetic nephropathy (DN) is the leading cause of chronic kidney disease worldwide and contributes to significant morbidity and mortality of diabetic patients. Approximately one-third of diabetic patients have progressive deterioration of renal function and ultimately require dialysis or transplantation [1]. This number is expected to rise dramatically as a result of the growing incidence of diabetes and the aging population [2,3]. The pathophysiological mechanisms of DN are incompletely understood, but numerous factors contribute to the pathogenesis and progression of DN. Transforming growth factor-β_1_ (TGF-β_1_) can induce the accumulation of extracellular matrix (ECM) components, including collagens, fibronectin (FN) and laminin in the glomeruli and the interstitium of the kidney. TGF-β_1_ expression regulates PINCH-1-integrin-linked kinase (ILK)-alpha-parvin complex formation and contributes to glomerular mesangial cell (GMC) proliferation and hypertrophy [4]. ILK, a cytoplasmic-binding serine/threonine protein kinase, is physically connected to the actin cytoskeleton and actin-binding protein CH-ILKBP, which is an important step in the development and progression of glomerular failure observed in several kidney diseases [5]. The GMCs, which have characteristics of a modified smooth muscle cell, occupy the central position in the renal glomerulus. The marker gene smooth muscle α-actin (α-SMA) was detected within the juxtamedullary glomeruli during foetal life. When glomerular development is completed after postnatal day 10, α-SMA expression is no longer present in the glomerulus [6]. GMCs can be activated by local injury, and activated cells are major sources of ECM synthesis, which affect the progression of renal dysfunction in human and experimental renal diseases [3,7].

Angiotensin II (Ang II) is considered to be involved in the majority of pathological processes that result in DN. Increased Ang II activity causes hypertrophy of GMCs. Ang II promotes the production of TGF-β_1_ that leads to progressive renal damage [8-10]. The death incidence due to cardiovascular disease is three times higher in patients with DN than in diabetic patients without signs of renal failure [11]. Because Ang II has an essential role in renal and cardiovascular pathophysiology, angiotensin-converting enzyme inhibitors (ACEI) have been shown to have beneficial effects on renal and cardiovascular diseases [11,12]. Benazepril, an ACEI, provides protection against the progressive deterioration of renal function in patients with renal diseases [13]. In this study, we investigate the effect of benazepril on the renal expression of TGF-β_1_, ILK and α-SMA in rat DN induced by streptozotocin (STZ) and the effect of benazepril on the expression of these genes associated with Ang II signalling pathway in GMCs. Our work demonstrates the renoprotective effects of benazepril *in vivo* and *in vitro*.

## Methods

### Animal model and glomerular isolation

Animal care methods and treatment were conducted in accordance with the guidelines of the Institutional Animal Care and Use Committee of Hebei Medical University and the study protocol was approved by the ethical committee of Hebei Medical University, Shijiazhuang, China (Additional file 1). Male Sprague–Dawley rats weighing 180–250 g were purchased from the Experimental Animal Academy of Chinese Medical Science Institute (Beijing, China). Diabetes was induced by a single tail-vein injection of STZ (Sigma-Aldrich, USA) at a dose of 65 mg/kg body wt; the STZ was freshly prepared in 0.1 mol/L citrate buffer (pH 4.5)[14]. Age-matched male non-diabetic control rats were injected with an equal volume of citrate buffer. Seventy-two hours after the STZ administration, the induction of diabetes was confirmed by measurement of the blood glucose concentration with the OneTouch II blood glucose meter (Johnson & Johnson, USA). The rats with blood glucose ≥16.7 mmol/L were considered to have diabetes. The rats were randomly divided into 3 groups: normal control group (NC, *n* = 12); diabetic nephropathy group (DN, *n* = 12); and diabetic nephropathy treated with benazepril (ACEI, *n* = 12). The diabetic rats were treated with benazepril (Beijing Novartis Pharmacy, China) at 10 mg/kg per day for 6 weeks by remedial perfusion of the stomach from the third day after the establishment of DN. All of the rats were kept individually in metabolic cages to collect 24-hour urine for the measurement of the 24-hour urinary protein (TP/24) at 8 weeks. Blood pressures (BP) were obtained using the Non-Invasive BP system (Kent Scientific Corp, Torrington, CT), and blood samples were collected from the inferior vena cava. The blood glucose (Glu), serum creatinine (Scr), and blood urea nitrogen (BUN) were measured as previously described[15]. The right kidney from each rat was dissected, rinsed with cold saline, placed in the Tissue-Tek O.C.T. compound (Sakura Finetek USA, Torrance, CA), snap frozen in liquid nitrogen and stored at −80°C until further analysis. The renal cortex of the left kidney from each rat was cut into small pieces, and the glomeruli were isolated by the mechanical graded sieving technique. After isolation, the purity of the final suspension was determined by phase contrast microscopy. On average, tubular contamination was less than 5%. The glomerular suspension was used to isolate proteins and RNA [14].

### Histological analysis, immunohistochemistry and immunofluorescent staining

Frozen sections (5 μm) of kidney were fixed and stained with haematoxylin and eosin (H & E) staining and periodic acid schiff (PAS) staining. For the immunohistochemistry staining, the frozen sections were fixed in pre-cooled acetone (−20°C) for 5 min. After being washed 3 times in PBS and treated with 0.3% H_2_O_2_ for 10 min, the slides were incubated with anti-CD68 (a marker gene for macrophage, #sc-9139), CD3ϵ (a marker gene for T lymphocyte, #sc-1127), and TGF-β_1_ antibodies (#sc-146, Santa Cruz Biotechnology, Santa Cruz, CA) overnight at 4°C. The slides were incubated with a biotinylated secondary antibody, which was followed by the Avidin Biotin Complex (ABC) Method for visualisation (R&D Systems, Minneapolis, MN). For glomerular assessment, mesangial area (percentage of glomerular positive stained) was quantitated from 10 glomeruli per kidney per animal using Image J (NIH, Bethesda, MD) [16]. For ILK and α-SMA immunofluorescent staining, the frozen sections were permeabilised with 0.1% Triton X-100 in PBS for 10 minutes and exposed to 1 × SuperBlock Reagent (Thermo scientific, Rockford, lL) with 5% normal goat serum for 1 hour. The sections were incubated with an anti-ILK antibody (1:200, #sc-13075, Santa Cruz Biotechnology, Santa Cruz, CA) or an anti-α-SMA antibody (1:500, #A2547, Sigma-Aldrich, St. Louis, MO) and then incubated with a Cy3 conjugated-goat anti-rabbit IgG secondary antibody (for ILK, #A10520) or an Alexa Fluor 488 conjugated goat anti mouse IgG secondary antibody (for α-SMA, #R37120, Life Technologies, Grand Island, NY). Images were acquired by laser-scanning confocal microscopy with 20× objectives (Zeiss LSM 510 meta) after being mounted with the ProLong gold anti-fade reagent with DAPI (Invitrogen, Carlsbad, CA).

### GMCs culture

GMCs were isolated from the glomerulus of four- to six-week-old Sprague–Dawley rats according to published methods [17,18] and were maintained in Dulbecco’s modified Eagle’s medium (DMEM, GIBCO, Carlsbad, CA) supplemented with 10% foetal bovine serum, 5.5 mmol/L glucose (normal glucose), 100 U/ml penicillin, 100 mg/ml streptomycin, 300 mg/ml L-glutamine at 37°C in a 5% CO_2_ incubator. To study the expression of TGF-β_1_, ILK and α-SMA, subconfluent GMCs were cultured in the presence of 5.5 mmol/L glucose (normal glucose group, NG), 5.5 mmol/L glucose plus 24.5 mmol/L mannitol (mannitol group, MG), 30 mmol/L glucose (high glucose group, HG), or 30 mmol/L glucose with benazepril 10 μmol/L (high glucose + benazepril group, ACEI) for periods of 3, 6, 12, 24, 48 and 72 h. The Ang II treatment was carried out in the four different types of medium used in the previous experiments for 32 h, and then serum starved with the same medium with the addition of 0.5% FBS for 16 h, followed by treatment with Ang II for 5 min (100 nM, Sigma-Aldrich, St. Louis, MO).

### RNA isolation and real-time quantitative RT-PCR

The total RNA was isolated from isolated glomeruli or GMCs with TRIzol (Invitrogen, Carlsbad, CA). The reverse transcriptase reactions were performed using the QuantiTect Reverse Transcription Kit (Qiagen, Valencia, CA). Real-time PCR was performed in triplicate on this cDNA using the ABI Prism 7700 sequence-detection system (Applied Biosystems, Foster City, CA). Designed Taqman® probes Mm01178820_m1, Mm01274281_g1 and Mm03944483_s1 for TGF-β_1_, ILK, and α-SMA were used following the manufacturer’s instructions (Applied Biosystems, Foster city, CA). The fold-change analysis was based on the normalised RNA levels by β-actin in the same sample.

### Western blotting

Tissue samples or cells were lysed in ice-cold RIPA buffer (150 mM NaCl, 50 mM Tris–HCl, pH 8.0, 1.0% NP-40, 0.5% sodium deoxycholate, 0.1% SDS) supplemented with a complete proteinase inhibitor (Roche Applied Sciences, Indianapolis, IN) and phosphatase inhibitor cocktails (Sigma-Aldrich, St. Louis, MO). Equal amounts of protein were resolved by SDS-PAGE and transferred to a PVDF membrane (BioRad, Hercules, CA) and blocked with anti-TGF-β_1_, ILK (Santa Cruz Biotechnology, Santa Cruz, CA), α-SMA (Sigma-Aldrich, St. Louis, MO), phospho-MAPK p44/42 (Thr^202^/Tyr^204^, #9101), total MAPK p44/42 (#9102), phospho-Akt (Ser^473^, #4060), and total Akt (#9272, Cell Signaling Technologies, Danvers, MA), followed by HRP–conjugated anti-rabbit or -mouse secondary antibodies (#7074 or #7076, Cell Signaling Technology, Danvers, MA). The blots were developed using an ECL system (PerkinElmer, Boston, MA). GAPDH (#2118, Cell Signaling Technology, Danvers, MA) was used as the loading control. The films were scanned and quantitative analysis of the ratio of phosphorylated to total MAPK p44/42, Akt or TGF-β_1_, ILK, and α-SMA from three independent experiments using Kodak 1D 3.5 software (Rochester, NY).

### Statistical analysis

All of the values are expressed as the mean ± S.E. The significance of the results was assessed by a 2-tailed non-parametric pair *t*-test (Mann–Whitney-U) or a two-way ANOVA with Bonferroni post hoc test (for >2 groups). *P* <0.05 was considered statistically significant.

## Results

### Body weight, systolic blood pressure and laboratory tests

As shown in Table 1, the body weight of DN rats was less than that of the control animals. The benazepril group grew significantly more than the animals with DN but slightly less than the normal counterparts. The DN rats developed mild hypertension, and a significantly increased BP was observed at 8 weeks in the rats. Benazepril treatment produced a significant decrease in the BP. The blood glucose levels of all of the DN groups were significantly higher than those of the control or benazepril treatment groups (*P* < 0.05). The blood glucose levels in the benazepril treatment group were lower than in the DN group (*P* < 0.05). As TP/24, Scr and BUN increased significantly in the DN group compared with the control group, Benazepril treatment significantly blunted this decrease (*P* < 0.05).

**Table 1:** Body weight, blood pressure, serum and urinary tests in different groups

**Group**	**BW (g)**	**BP (mmHg)**	**Glu (mmol/L)**	**TP/24 (ml)**	**Scr (μmol/L)**	**Bun (mmol/L)**
NC	227.0 ± 21.8	105.3 ± 6.8	6.97 ± 0.81	6.82 ± 1.97	48.32 ± 2.37	7.99 ± 0.58
DN	158.4 ± 9.3*	142.4 ± 5.1*	35.63 ± 2.13*	25.23 ± 4.23*	72.85 ± 4.97*	18.32 ± 1.96*
ACEI	187.6 ± 9.1*#	123.4 ± 4.4*#	21.71 ± 3.87*#	15.12 ± 2.43*#	57.34 ± 3.79*#	12.02 ± 1.09*#

Values are shown as the mean ± S.E. *,*P* < 0.05 vs. control; #,*P* < 0.05 vs. DN.

### Renal histology

**Figure 1:**
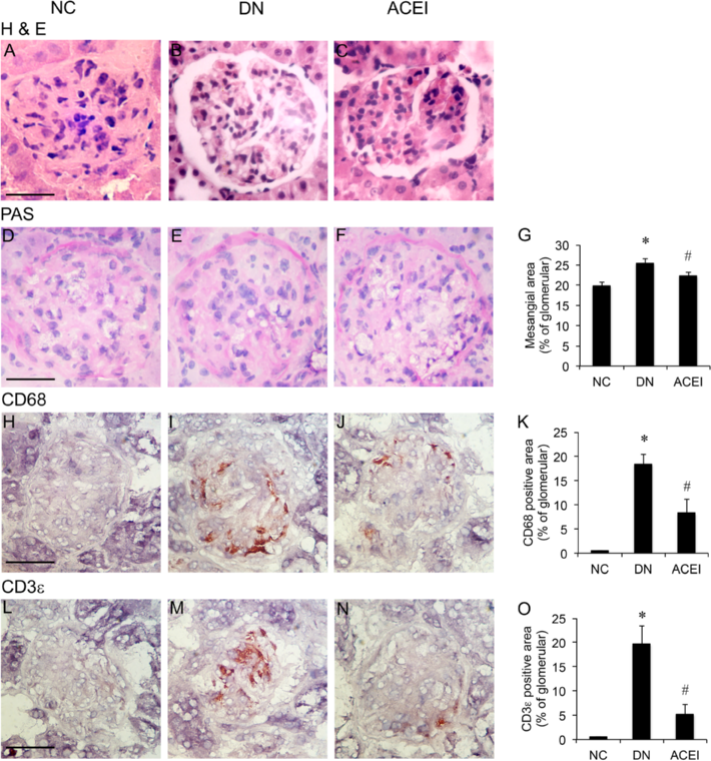
**Renal histology. (A-C)** H&E stained kidney sections of the normal control (NC), diabetic nephropathy group (DN), and diabetic nephropathy treated with benazepril (ACEI). DN and ACEI groups showing the typical structures of the glomeruli and tubules. **(D-F)** Periodic acid-Schiff (PAS) staining was performed in the kidneys of the different groups. **(G)** Quantification of glomerular PAS staining. **(H-J)** CD68 immunohistochemistry staining in the kidneys of the different groups. **(K)** Quantification of glomerular staining for CD68. **(L-N)** CD3ϵ immunohistochemistry staining in the kidneys of the different groups. **(O)** Quantification of glomerular staining for CD3ϵ. An increase in mesangial area was observed in DN rat mice as compared with control rat. The ACEI group that treated with Benazepril showed less inflammatory cells, less thickening of basement membrane and capillaries. *n* = 12. Bars = 50 μm. *, *P* < 0.05 vs. control; #, *P* < 0.05 vs. DN.

We compared the renal histology among the 3 groups. H & E staining and PAS staining of the kidney from the control group revealed a normal glomerulus surrounded by Bowman’s capsule and proximal and distal convoluted tubules without any inflammatory changes. The DN rats showed renal tubule atrophy (Figure 1A-C), thickening of the basement membrane (Figure 1D-G) and degenerated glomeruli infiltrated by inflammatory cells (Figure 1H-O). The ACEI group showed significatly fewer inflammatory cells, reduced thickening of the basement membrane and capillaries compared with DN group (Figure 1A-O).

### Benazepril affects TGF-β_1_, ILK and α-SMA expression in renal tissue

**Figure 2:**
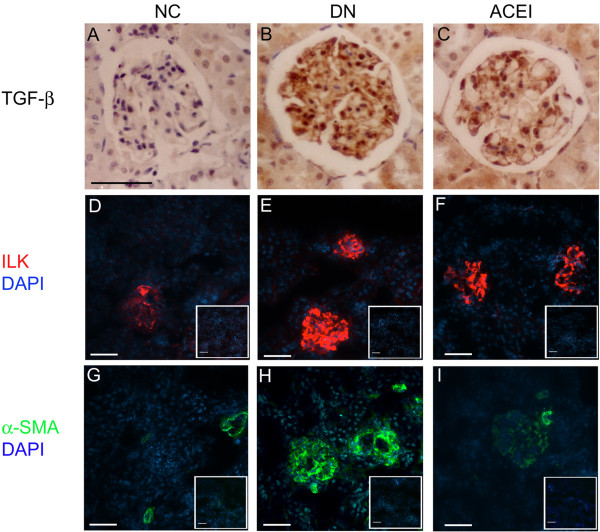
**Benazepril affects the TGF-β**_**1**_**, ILK and α-SMA expression in renal tissue and glomeruli. (A-C)** Immunohistochemistry staining of TGF-β_1_ in the rat kidneys of the NC, DN and ACEI groups. **(D-F)** Immunofluorescence staining of ILK in rat kidneys of NC, DN and ACEI group. There are significant increases in ILK expression in DN rat compared with control mice. Rabbit IgG was used as a negative control and shown in the right corner of each figure. **(G-I)** Immunofluorescence staining of α-SMA in the rat kidneys of the different groups. There are significant increases in α-SMA expression in DN rat compared with control mice. Mouse IgG was used as a negative control and is shown in the right corner of each figure. Bars = 50 μm.

To further examine the development of the histological changes in diabetes, we examined the molecular pathology that is classically associated with DN. TGF-β_1_ expression has emerged as a key participant in the cascade of events that leads to DN [19]. Immunohistochemistry staining showed that the basal level of TGF-β_1_ expression was barely detectable in the control rat kidney (Figure 2A). The DN group showed strongly positive TGF-β_1_ expression, mainly distributed in the glomerulus, GMCs, and tubular interstitium(Figure 2B). Benazepril treatment significantly decreased the TGF-β_1_ expression in the renal tissue (Figure 2C). Immunofluorescence staining for ILK and α-SMA expression, paralleled with that for TGF-β_1_, was faintly positive (ILK, Figure 2D) or undetectable (α-SMA, Figure 2G) in the rat renal glomerulus and the GMCs in the control group. Strongly positive staining was observed in the rat glomerulus and the GMCs in the DN group (ILK, Figure 2E; α-SMA, Figure 2H). Benazepril treatment significantly decreased the expression of ILK and α-SMA in the rat glomerulus (Figure 2F and I), suggesting that the beneficial effects of benazepril were mainly mediated by the inhibition of TGF-β_1_, ILK and α-SMA expression in diabetic renal tissue.

### Benazepril affects TGF-β_1_, ILK and α-SMA expression in glomeruli

**Figure 3:**
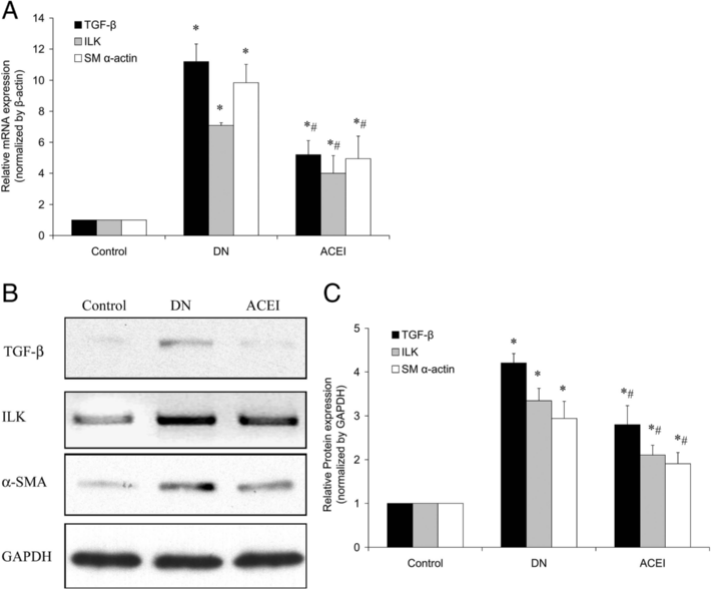
**Benazepril affects TGF-β**_**1**_**, ILK and α-SMA mRNA and protein expression in glomeruli. (A)** TGF-β_1_, ILK and α-SMA mRNA expression levels in the glomeruli of the NC, DN and ACEI group were determined by real-time RT-PCR. **(B)** ILK and α-SMA protein expression levels in the glomeruli of the different groups were determined by western blot. **(C)** Representative western blot analysis. Densitometric quantification of the corresponding bands is performed using Kodak 1D Image software. The values are the means ± SE and are expressed relative to the control. *n* = 12. *, *P* < 0.05 vs. control; #, *P* < 0.05 vs. DN.

TGF-β_1_, ILK and α-SMA mRNA expression were determined by real-time RT-PCR (Figure 3A). The expression levels of all of the examined molecules were higher in the diabetic glomeruli than in the control glomeruli. Benazepril treatment inhibited the expression of those molecules, but failed to rescue to the same level in the normal group. The western blot analysis showed a similar pattern of expression (Figure 3B and C). The control glomeruli showed a faint band of the ILK or α-SMA protein, while the bands were stronger in the diabetic glomeruli. The bands in the benazepril treatment group were lower than in the DN group but higher than in the normal group (Figure 3B and C).

### Benazepril affects TGF-β_1_, ILK and α-SMA expression in cultured GMCs

**Figure 4:**
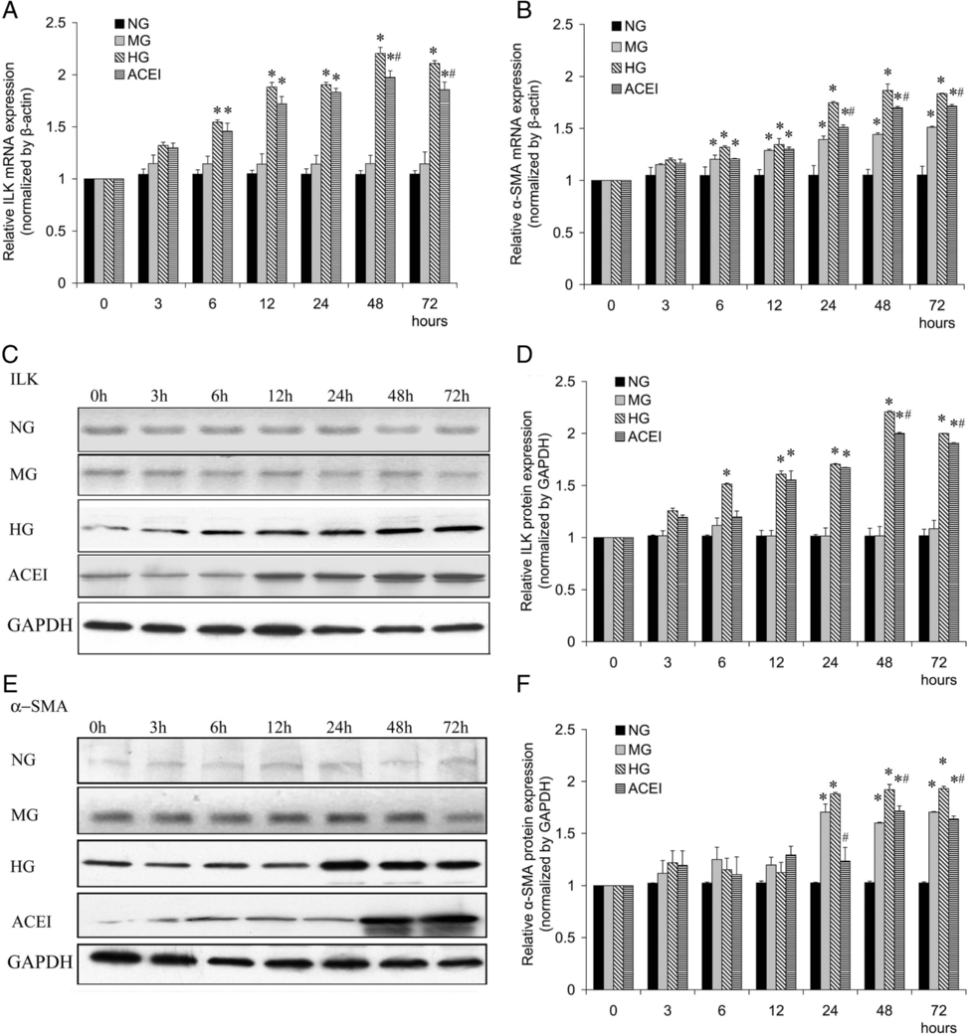
**Benazepril affects the TGF-β**_**1**_**, ILK and α-SMA expression in glomerular mesangial cell (GMC).** Benazepril affects the expression of ILK **(A)** and α-SMA **(B)** mRNA in GMCs under HG conditions at different times, as determined by real-time RT-PCR. Benazepril affects the expression of ILK (**C** and **D**) and α-SMA (**E** and **F**) proteins in GMCs under HG conditions at different time points, as determined by western blot analysis. Densitometric quantification of the corresponding bands is performed using Kodak 1D Image software. The values are the means ± SE and are expressed relative to the control. *n* = 6. *, *P* < 0.05 vs. control; #, *P* < 0.05 vs. DN.

The RT-PCR results showed that HG levels induced ILK mRNA in a time-dependent manner compared with the NG. The ILK mRNA expression began to increase at 3 h and reached a peak at 48 h after treatment. The ILK mRNA expression began to decrease at 72 h but remained higher than the levels in the NG and HG at each time point (Figure 4A). Benazepril treatment did not completely attenuate the HG induced ILK mRNA expression at 48 h or 72 h, and the levels remained higher than in the NG. There were no significant differences between the MG and NG groups. The expression of α-SMA mRNA showed a similar pattern (Figure 4B). The western blotting analysis revealed that ILK and α-SMA protein expression in the HG started to increase as early as 6 h (ILK, *P* < 0.05) or 24 h (α-SMA, *P* < 0.05) and reached a peak at 48 h (Figure 4 C-F). Benazepril treatment inhibited the HG induced ILK and α-SMA protein expression in the ACEI group at 48 h, but it remained higher than in the NG or MG (Figure 4 C-F). We found that the expression levels of α-SMA mRNA and protein in the MG were increased as early as 6 h (mRNA, Figure 4B) or 24 h (Protein, Figure 4E and F), which was higher than in the NG (*P* < 0.05), suggesting that high osmotic pressure might cause the increase in the α-SMA expression [20].

**Figure 5:**
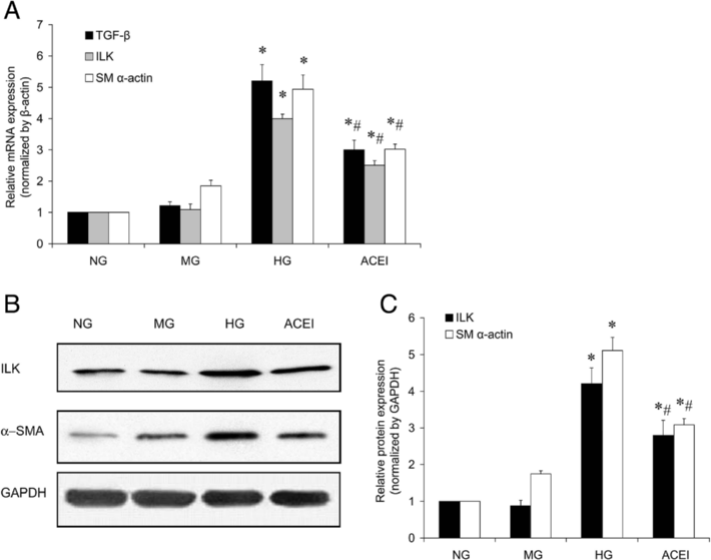
**Benazepril treatment inhibited the high glucose induced TGF-β**_**1**_**, ILK and α-SMA expression at 48 h. (A)** The TGF-β_1_, ILK and α-SMA mRNA expression in NG, MG, HG and ACEI were determined by real-time RT-PCR at 48 h. **(B)** Western blotting determined ILK and α-SMA protein expression in the NG, MG, HG and ACEI at 48 h. **(C)** A representative western blot analysis. Densitometric quantification of the corresponding bands is performed using Kodak 1D Image software. Values are means ± SE and are expressed relative to the control. *n* = 6. *,*P* < 0.05 vs control; #, *P* < 0.05 vs DN.

To confirm the effect of benazepril on the TGF-β_1_ mRNA expression and ILK and α-SMA protein synthesis, RNA and protein were isolated from GMCs after being cultured in NG, MG, HG, or ACEI for 48 h. The results showed that HG levels upregulated TGF-β_1_ mRNA expression, and benazepril treatment effectively blocked the HG induced TGF-β_1_ mRNA expression (Figure 5A). The western blot analysis showed that the HG levels increased the ILK and α-SMA expression, parallel with TGF-β_1_ mRNA expression in response to Benazepril, suggesting that the expression of those molecules was affected by HG induced TGF-β_1_ expression (Figure 5B and C).

### Benazepril affects Ang II signalling pathways in GMCs

**Figure 6:**
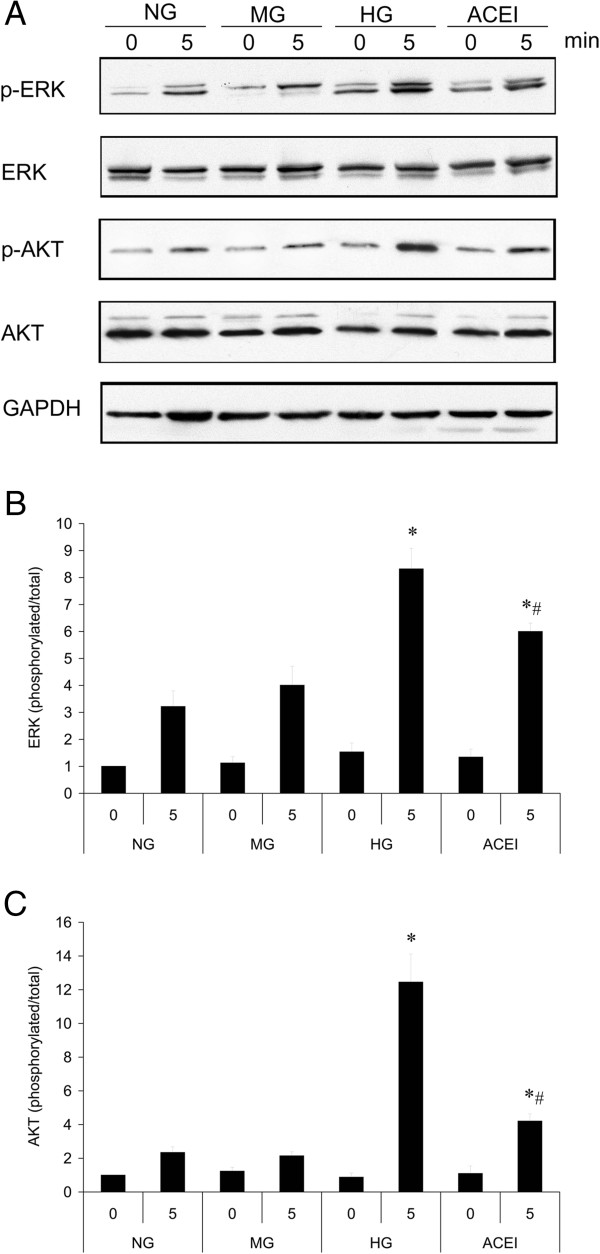
**Benazepril affects Ang II signal pathways in cultured glomerular mesangial cell (GMC). (A)** Ang II-induced ERK Thr^202^/Tyr^204^ and Akt Ser^473^ phosphorylation in the NG, MG, HG and ACEI were determined by western blotting. Representation of the quantitative analysis of the ratio of the phosphorylated/total ERK **(B)** or Akt **(C)** from three independent experiments. Densitometric quantification of the corresponding bands is performed using Kodak 1D Image software. The values are the means ± SE and are expressed relative to the control. *n* = 3. *, *P* < 0.05 vs. control; #, *P* < 0.05 vs. DN.

High-glucose exposure can increase Ang II generation in cultured GMCs [21]. To elucidate the mechanism(s) of the effect of benazepril on GMC cellular processes, we assessed the effect of benazepril on Ang II signalling pathways. ERK activation supports GMC proliferation, while Akt phosphorylation mediates cell growth and TGF-β gene transcription [22]. Western blotting showed that HG levels enhanced Ang II-induced ERK and Akt phosphorylation in GMCs (Figure 6A-C). Mannitol did not have a significant induction effect on the basal phosphorylation of ERK or Akt at the same time point. Benazepril significantly attenuated the HG induced ERK and Akt phosphorylation, indicating that benazepril affected the GMC function through Ang II signalling in the GMCs (Figure 6A-C).

## Discussion

DN is a common cause of end-stage kidney disease worldwide. The characteristic early abnormalities of diabetic kidneys are increased renal size and hyperfiltration. With the alteration of the glomerular filtration barrier, the glomerular structure collapses and leads to an increase in the albumin excretion rate followed by the development of GMC proliferation, ECM accumulation, and glomerular sclerosis. GMC proliferation is often considered an initial, adaptive response that eventually loses control and develops into a pathological process [23,24]. HG induced autocrine or paracrine variety growth factors, cytokines, chemokines and vasoactive agents, including TGF-β_1_ and Ang II, have been implicated in the stimulation of ECM accumulation following structural changes of DN. TGF-β_1_ expression was increased in experimental diabetic animals and diabetic patients. Anti-TGF-β_1_ antibody or TGF-β_1_ antisense oligonucleotides attenuated renal hypertrophy or HG induced GMC FN expression by inhibition of ECM gene expression. Ang II can induce TGF-β_1_ expression in GMCs, suggesting that TGF-β_1_ is the final common mediator of DN [10,25,26]. ILK plays an important role in the interface between TGF-β_1_, ECM, the actin-based cytoskeleton and the cellular phenotype in kidney diseases [27]. We determined that ILK expression increased in the renal tissue of DN rats or in HG treated GMCs, indicating that HG levels induced ILK expression at least in part through increasing TGF-β_1_ autocrine secretion. Benazepril could attenuate the HG level induced TGF-β_1_ and ILK expression *in vivo* or *in vitro*, suggesting that Ang II also affects TGF-β_1_ and ILK expression.

GMCs that are activated by local injury impaired the activation of α-SMA expression following GMC proliferation and basement membrane remodelling, which affects the glomerular filtration. Diabetes was shown to produce an increase in the expression of α-SMA in the kidney glomeruli and to result in the accumulation of type IV collagen, resulting in renal fibrosis and nephropathy [6,28]. We observed that α-SMA expression was significantly increased in the DN rat, and HG levels increased the α-SMA mRNA and protein levels in GMCs with a time-dependent pattern. The increased α-SMA expression was an important step in the GMC phenotypic changes from the non-activated state to the proliferative, secretory activated state. Activated GMCs increased ECM production, increased inflammatory response, increased their own proliferation, and lead to glomerulosclerosis [29]. HG levels induced Ang II generation in cultured GMCs [21]. Ang II increases vascular resistance, reduces renal blood flow, and stimulates the production of ECM in the GMCs, which is one of the abnormalities in diabetic renal disease [9,30]. The ERK pathway was shown to be crucial in cell proliferation and differentiation, which is considered to be an important molecular mechanism in the development and progression of DN [31,32]. The Akt pathway is a critical regulator for a variety of cellular processes, including cell growth, cell motility, and TGF-β_1_ gene transcription in GMCs [33,34]. We determined that HG levels enhanced Ang II-induced ERK and Akt phosphorylation in GMCs. Benazepril significantly attenuated the HG induced ERK and Akt phosphorylation. We found TGF-β_1_ expression increased in the glomeruli of diabetic rats and in cultured GMCs under HG.

Benazepril, a kind of multifunction drug, primarily used in treatment of hypertension, congestive heart failure, and heart attacks, and also has beneficial effects in preventing renal and retinal complications of diabetes [35]. ACEI treatment showed lower in body weight, lower blood pressure, and a bit unexpectedly lower blood glucose levels than DN group (Table 1). ACE-I treated group was really specific to the inhibition of the renin-angiotensin-aldosterone system rather than secondary to i) lower body weight, thus less hyperfiltration and less glomerular hypertrophy, ii) less hyperglycemia and most importantly iii) lower blood pressure. All of these factors are well known driving factors behind the development of DN. For the therapeutic effect, ACEI group, the real control of DN group, showed the renoprotective function in the development of DN, but still a diabetic group treated with e.g. a thiazide diuretic to lower blood pressure level to a similar degree as seen in the treatment group should be used in the future, which it helps a great deal to illustrate that our results remain consistent across the data in vivo and in vitro.

## Conclusions

ERK and Akt play roles in the development and progression of DN, and they might be potential therapeutic targets. The findings described here support the hypothesis that the high-glucose milieu of diabetes increases TGF-β_1_ secretion, which increases the synthesis of ILK and α-SMA that are involved in the progression of DN. This might be an important mechanism of the benazepril renoprotective function in the pathogenesis of DN.

## Abbreviations

DN: Diabetic nephropathy; ACEI: Angiotensin converting enzyme inhibitor; TGF-β_1_: Transforming growth factor-β_1_; ILK: Integrin-linked kinase; GMC: Glomerular mesangial cell; FN: Fibronectin; α-SMA: Smooth muscle α-actin; Ang II: Angiotensin II; ECM: Extracellular matrix.

## Competing interests

The authors declare that they have no competing interests.

## Authors’ contributions

HLN performed the experiments and data analysis, participated in the design of the dtudy and drafted the manuscript. LN participated in its design and coordination and helped to draft the manuscript. MDL contributed to the design of the sutdy and helped wth perfoming eht experiments and drafting the manuscripe. YQC and TZ provided intellectual imput to the study and helped with the revision of the manuscript. YL conceived of and designed the study and supervided the work. All authors read and approved the final manuscript.

## Pre-publication history

The pre-publication history for this paper can be accessed here:

http://www.biomedcentral.com/1471-2369/15/135/prepub

## Supplementary Material

Additional file 1 The ARRIVE Guidelines Checklist. Click here for file
