# Supplementary material for: Benazepril affects integrin-linked kinase and smooth muscle α-actin expression in diabetic rat glomerulus and cultured mesangial cells
Source: BMC Nephrol. 2014 Aug 20;15:135. doi: 10.1186/1471-2369-15-135 (PMC4151867; doi:10.1186/1471-2369-15-135)
Supplement: Additional file 1 — The ARRIVE Guidelines Checklist. [file 1471-2369-15-135-S1.pdf]

ARRIVE

The ARRIVE Guidelines Checklist

Animal Research: Reporting In Vivo Experiments

Carol Kilkenny[1], William J Browne[2], Innes C Cuthill[3], Michael Emerson[4] and Douglas G Altman[5]

[1]The National Centre for the Replacement, Refinement and Reduction of Animals in Research, London, UK, [2]School of Veterinary Science, University of Bristol, Bristol, UK, [3]School of Biological Sciences, University of Bristol, Bristol, UK, [4]National Heart and Lung Institute, Imperial College London, UK, [5]Centre for Statistics in Medicine, University of Oxford, Oxford, UK.

	ITEM	RECOMMENDATION	Section/ Paragraph
Title	1	Provide as accurate and concise a description of the content of the article as possible.	Benazepril affects
Abstract	2	Provide an accurate summary of the background, research objectives, including details of the species or strain of animal used, key methods, principal findings and conclusions of the study.	Background: Diabetic nephropathy
**INTRODUCTION**			
Background	3	a. Include sufficient scientific background (including relevant references to previous work) to understand the motivation and context for the study, and explain the experimental approach and rationale.<br>b. Explain how and why the animal species and model being used can address the scientific objectives and, where appropriate, the study's relevance to human biology.	a. Diabetic nephropathy (DN) is the leading cause of chronic [illegible]
Objectives	4	Clearly describe the primary and any secondary objectives of the study, or specific hypotheses being tested.	The objectives of
**METHODS**			
Ethical statement	5	Indicate the nature of the ethical review permissions, relevant licences (e.g. Animal [Scientific Procedures] Act 1986), and national or institutional guidelines for the care and use of animals, that cover the research.	Animal care methods and treatment
Study design	6	For each experiment, give brief details of the study design including:<br>a. The number of experimental and control groups.<br>b. Any steps taken to minimise the effects of subjective bias when allocating animals to treatment (e.g. randomisation procedure) and when assessing results (e.g. if done, describe who was blinded and when).<br>c. The experimental unit (e.g. a single animal, group or cage of animals).<br>A time-line diagram or flow chart can be useful to illustrate how complex study designs were carried out.	a. Three groups of 12 rat each were studied. Male Sprague-Daw ley rats weighing 180-250 g were
Experimental procedures	7	For each experiment and each experimental group, including controls, provide precise details of all procedures carried out. For example:<br>a. How (e.g. drug formulation and dose, site and route of administration, anaesthesia and analgesia used [including monitoring], surgical procedure, method of euthanasia). Provide details of any specialist equipment used, including supplier(s).<br>b. When (e.g. time of day).<br>c. Where (e.g. home cage, laboratory, water maze).<br>d. Why (e.g. rationale for choice of specific anaesthetic, route of administration, drug dose used).	a. Diabetes was induced by a single tail-vein injection of STZ (Sigma-Aldric h, USA) at a dose of 65 mg/kg body wt; the STZ
Experimental animals	8	a. Provide details of the animals used, including species, strain, sex, developmental stage (e.g. mean or median age plus age range) and weight (e.g. mean or median weight plus weight range).<br>b. Provide further relevant information such as the source of animals, international strain nomenclature, genetic modification status (e.g. knock-out or transgenic), genotype, health/immune status, drug or test naïve, previous procedures, etc.	a. Male Sprague-Daw ley rats weighing 180-250 g<br><br>b. Thirty-six

Housing and husbandry	9	Provide details of:<br>a. Housing (type of facility e.g. specific pathogen free [SPF]; type of cage or housing; bedding material; number of cage companions; tank shape and material etc. for fish).<br>b. Husbandry conditions (e.g. breeding programme, light/dark cycle, temperature, quality of water etc for fish, type of food, access to food and water, environmental enrichment).<br>c. Welfare-related assessments and interventions that were carried out prior to, during, or after the experiment.	a. Animals were housed with a 12 light/12 dark cycle in a temperature and humidity controlled room.
Sample size	10	a. Specify the total number of animals used in each experiment, and the number of animals in each experimental group.<br>b. Explain how the number of animals was arrived at. Provide details of any sample size calculation used.<br>c. Indicate the number of independent replications of each experiment, if relevant.	a. Thirty-six rats were randomly divided into 3 groups: normal
Allocating animals to experimental groups	11	a. Give full details of how animals were allocated to experimental groups, including randomisation or matching if done.<br>b. Describe the order in which the animals in the different experimental groups were treated and assessed.	a. For experiments, rats were randomly
Experimental outcomes	12	Clearly define the primary and secondary experimental outcomes assessed (e.g. cell death, molecular markers, behavioural changes).	Two primary outcome
Statistical methods	13	a. Provide details of the statistical methods used for each analysis.<br>b. Specify the unit of analysis for each dataset (e.g. single animal, group of animals, single neuron).<br>c. Describe any methods used to assess whether the data met the assumptions of the statistical approach.	a. All of the values are expressed as the mean ±S.E. The
RESULTS			
Baseline data	14	For each experimental group, report relevant characteristics and health status of animals (e.g. weight, microbiological status, and drug or test naïve) prior to treatment or testing. (This information can often be tabulated).	The animals' health status was
Numbers analysed	15	a. Report the number of animals in each group included in each analysis. Report absolute numbers (e.g. 10/20, not 50%[2]).<br>b. If any animals or data were not included in the analysis, explain why.	Twelve animals in each group
Outcomes and estimation	16	Report the results for each analysis carried out, with a measure of precision (e.g. standard error or confidence interval).	In accordance with the
Adverse events	17	a. Give details of all important adverse events in each experimental group.<br>b. Describe any modifications to the experimental protocols made to reduce adverse events.	a. We did not found any important
DISCUSSION			
Interpretation/ scientific implications	18	a. Interpret the results, taking into account the study objectives and hypotheses, current theory and other relevant studies in the literature.<br>b. Comment on the study limitations including any potential sources of bias, any limitations of the animal model, and the imprecision associated with the results[2].<br>c. Describe any implications of your experimental methods or findings for the replacement, refinement or reduction (the 3Rs) of the use of animals in research.	a. The aim of our study was to discuss the Benazepril beneficial effects in preventing renal and
Generalisability/ translation	19	Comment on whether, and how, the findings of this study are likely to translate to other species or systems, including any relevance to human biology.	The findings described here support
Funding	20	List all funding sources (including grant number) and the role of the funder(s) in the study.	This study was

References:
1. Kilkenny C, Browne WJ, Cuthill IC, Emerson M, Altman DG (2010) Improving Bioscience Research Reporting: The ARRIVE Guidelines for Reporting Animal Research. *PLoS Biol* 8(6): e1000412. doi:10.1371/journal.pbio.1000412
2. Schulz KF, Altman DG, Moher D, the CONSORT Group (2010) CONSORT 2010 Statement: updated guidelines for reporting parallel group randomised trials. *BMJ* 340:c332.

1. Remuzzi G, Schieppati A, Ruggenenti P: Clinical practice. Nephropathy in patients with type 2 diabetes. N Engl J Med 2002, 346(15):1145–1151.
2. Sego S: Pathophysiology of diabetic nephropathy. Nephrol Nurs J 2007, 34(6):631–633.
3. Kanwar YS, Wada J, Sun L, Xie P, Wallner EI, Chen S, Chugh S, Danesh FR: Diabetic nephropathy: mechanisms of renal disease progression. Exp Biol Med (Maywood) 2008, 233(1):4–11.
4. Kim SM, Kim N, Lee S, Kim do K, Lee YM, Ahn SH, Song JH, Choi BK, Wu C, Jung KY: TGF-beta1-induced PINCH-1-ILK-alpha-parvin complex formation regulates mesangial cell proliferation and hypertrophy. Exp Mol Med 2007, 39(4):514–523.
5. Guo L, Sanders PW, Woods A, Wu C: The distribution and regulation of integrin-linked kinase in normal and diabetic kidneys. Am J Pathol 2001, 159(5):1735–1742.
6. Carey AV, Carey RM, Gomez RA: Expression of alpha-smooth muscle actin in the developing kidney vasculature. Hypertension 1992, 19(2 Suppl):II168–175.
7. Hu P, Qin YH, Pei J, Lei FY, Hu B, Lu L: Beneficial effect of all-trans retinoic acid (ATRA) on glomerulosclerosis rats via the down-regulation of the expression of alpha-smooth muscle actin: a comparative study between ATRA and benazepril. Exp Mol Pathol 2010, 89(1):51–57.
8. Jacobson HR: Chronic renal failure: pathophysiology. Lancet 1991, 338(8764):419–423.
9. Leehey DJ, Singh AK, Alavi N, Singh R: Role of angiotensin II in diabetic nephropathy. Kidney Int Suppl 2000, 77:S93–98.
10. Border WA, Noble NA: Evidence that TGF-beta should be a therapeutic target in diabetic nephropathy. Kidney Int 1998, 54(4):1390–1391.
11. Parving HH: Diabetic nephropathy: prevention and treatment. Kidney Int 2001, 60(5):2041–2055.
12. Jacobsen P, Rossing K, Parving HH: Single versus dual blockade of the renin-angiotensin system (angiotensin-converting enzyme inhibitors and/or angiotensin II receptor blockers) in diabetic nephropathy. Curr Opin Nephrol Hypertens 2004, 13(3):319–324.
13. Maschio G, Alberti D, Janin G, Locatelli F, Mann JF, Motolese M, Ponticelli C, Ritz E, Zucchelli P: Effect of the angiotensin-converting-enzyme inhibitor benazepril on the progression of chronic renal insufficiency. The Angiotensin-Converting-Enzyme Inhibition in Progressive Renal Insufficiency Study Group. N Engl J Med 1996, 334(15):939–945.
14. Satoh M, Fujimoto S, Haruna Y, Arakawa S, Horike H, Komai N, Sasaki T, Tsujioka K, Makino H, Kashihara N: NAD(P)H oxidase and uncoupled nitric oxide synthase are major sources of glomerular superoxide in rats with experimental diabetic nephropathy. Am J Physiol Renal Physiol 2005, 288(6):F1144–1152.
15. Parasuraman S, Raveendran R: Measurement of invasive blood pressure in rats. J Pharmacol Pharmacother 2012, 3(2):172–177.
16. Watson AM, Gray SP, Jiaze L, Soro-Paavonen A, Wong B, Cooper ME, Bierhaus A, Pickering R, Tikellis C, Tsorotes D, Thomas MC, Jandeleit-Dahm KA: Alagebrium reduces glomerular fibrogenesis and inflammation beyond preventing RAGE activation in diabetic apolipoprotein E knockout mice. Diabetes 2012, 61(8):2105–2113.
17. Okuda S, Languino LR, Ruoslahti E, Border WA: Elevated expression of transforming growth factor-beta and proteoglycan production in experimental glomerulonephritis. Possible role in expansion of the mesangial extracellular matrix. J Clin Invest 1990, 86(2):453–462.
18. Huang H, Li Y, Liu M, Shi Y, Zhang T: Gremlin induces cell proliferation and extra cellular matrix accumulation in mouse mesangial cells exposed to high glucose via the ERK1/2 pathway. BMC Nephrol 2013, 14(33):1471–2369.
19. Border WA, Ruoslahti E: Transforming growth factor-beta in disease: the dark side of tissue repair. J Clin Invest 1992, 90(1):1–7.
20. Sheetz MJ, King GL: Molecular understanding of hyperglycemia's adverse effects for diabetic complications. Jama 2002, 288(20):2579–2588.
21. Vidotti DB, Casarini DE, Cristovam PC, Leite CA, Schor N, Boim MA: High glucose concentration stimulates intracellular renin activity and angiotensin II generation in rat mesangial cells. Am J Physiol Renal Physiol 2004, 286(6):F1039–1045.
22. Mondorf UF, Geiger H, Herrero M, Zeuzem S, Piiper A: Involvement of the platelet-derived growth factor receptor in angiotensin II-induced activation of extracellular regulated kinases 1 and 2 in human mesangial cells. FEBS Lett 2000, 472(1):129–132.
23. Wesson LG: Physical factors and glomerulosclerosis. Cause or coincidence? Nephron 1998, 78(2):125–130.
24. Lopez-Novoa JM, Rodriguez-Pena AB, Ortiz A, Martinez-Salgado C, Lopez Hernandez FJ: Etiopathology of chronic tubular, glomerular and renovascular nephropathies: clinical implications. J Transl Med 2011, 9:13.
25. Kolm V, Sauer U, Olgemoller B, Schleicher ED: High glucose-induced TGF-beta 1 regulates mesangial production of heparan sulfate proteoglycan. Am J Physiol 1996, 270(5 Pt 2):F812–821.
26. Oh JH, Ha H, Yu MR, Lee HB: Sequential effects of high glucose on mesangial cell transforming growth factor-beta 1 and fibronectin synthesis. Kidney Int 1998, 54(6):1872–1878.
27. Blattner SM, Kretzler M: Integrin-linked kinase in renal disease: connecting cell-matrix interaction to the cytoskeleton. Curr Opin Nephrol Hypertens 2005, 14(4):404–410.
